# Prevalence, characteristics, and surgical outcomes of cardiac masses: a 6-year retrospective cohort study

**DOI:** 10.1186/s12872-026-05917-3

**Published:** 2026-04-29

**Authors:** Mohsen Mir Mohammadsadeghi, Babak Darakhshandeh, Marjan Jamalian, Ehsan Shirvani, Nazanin Soleimani, Sana Azizian

**Affiliations:** 1 Chamran Cardiovascular Medical and Research Hospital, Department of Cardiac Surgery, Isfahan University of Medical Sciences, Isfahan, Iran; 2 Chamran Cardiovascular Medical and Research Hospital, Isfahan University of Medical Sciences, Isfahan, Iran; 3 Interventional Cardiology Research Center, Cardiovascular Research Institute, Isfahan University of Medical Sciences, Isfahan, Iran; 4 Cardiac Rehabilitation Research Center, Cardiovascular Research Institute, Isfahan University of Medical Sciences, Isfahan, Iran

**Keywords:** Cardiac neoplasms, Echocardiography, Surgical resection, Postoperative outcomes, Retrospective cohort study

## Abstract

**Background:**

Cardiac masses (CMs) pose a significant diagnostic challenge. Echocardiography is the primary modality for both initial evaluation and perioperative assessment due to its accessibility and ability to assess cardiac structure and function. However, data on postoperative echocardiographic changes remain limited. Also, comprehensive regional data, particularly from Iran, are scarce, and studies evaluating paired pre- and postoperative echocardiographic parameters are lacking. Accordingly, this study aimed to evaluate echocardiographic changes following surgical resection and to characterize the clinical and histopathological features of CMs in a regional cohort.

**Methods:**

This retrospective cohort study (2018–2023) included 75 patients with surgically resected CMs from two tertiary centers. Diagnoses were confirmed by transthoracic echocardiography and histopathology. Pre- and postoperative echocardiographic parameters were compared, and clinical and demographic data were obtained from medical records. Paired statistical tests were used, with *p* < 0.05 considered significant.

**Results:**

Among 75 patients (mean age 53.6 ± 17.4 years; 50.7% male), most had benign tumors (69.3%), predominantly myxomas (52%). The left atrium was the most common location (58.2%). A significant postoperative change was observed in right ventricular size distribution (*p* = 0.008), while other echocardiographic parameters remained unchanged.

**Conclusion:**

Surgical resection of CMs was not associated with significant short-term functional impairment. These findings highlight the value of postoperative echocardiographic assessment and the need for further studies with early and long-term follow-up. This study provides novel regional data from Iran and supports the need for larger prospective studies to validate these findings.

## Background

Cardiac masses (CMs) are broadly classified into primary benign tumors, primary malignant tumors, secondary (metastatic) malignancies, and pseudotumors [1]. Secondary tumors are 20–40 times more common than primary tumors [2]. Most PCTsare benign (75%–90%), with myxomas comprising over half, while sarcomas are the most common malignant primary tumors [3–5]. Pseudotumors are also common and include a wide range of entities, from normal anatomical variants to pathological conditions such as thrombi [6].

Although cardiac tumors are rare, the increasing use of echocardiography and advances in imaging have improved the detection of CMs [7]. Clinical presentation varies widely depending on tumor size, location, and growth rate, with many cases remaining asymptomatic and incidentally detected [8–10]. When present, symptoms are often nonspecific and include systemic manifestations, cardiac symptoms, embolic events, and immunologic features which may contribute to delayed diagnosis [8, 11].

Echocardiography remains the primary and most accessible imaging modality for the initial evaluation of CMs [12]; however, despite its widespread use and high diagnostic accuracy as a first-line tool, it has inherent limitations in distinguishing among various CM types. Consequently, standardized diagnostic algorithms remain insufficiently established [9, 13]. Cardiac magnetic resonance (CMR) imaging is therefore considered the noninvasive gold standard for detailed tissue characterization and comprehensive assessment [14].

The diagnostic workup generally begins with chest radiography, followed by transthoracic or transesophageal echocardiography, and may be supplemented with computed tomography and CMR for further evaluation [4, 8, 9, 15].

The prognosis of cardiac tumors depends on their type, location, and treatment strategy [16]. Benign primary cardiac tumors (PCT) generally have favorable outcomes after surgical resection, although delayed treatment may lead to significant morbidityn contrast, malignant PCTs are characterized by aggressive behavior, including local invasion, metastasis, high recurrence rates, and poor survival despite treatment [4, 5, 17]. Similarly, metastatic cardiac tumors carry a poor prognosis, largely determined by the primary malignancy and disease extent [4, 18–20].

Surgical resection remains the cornerstone of treatment and is often curative in benign tumors, whereas malignant tumors typically require multimodal therapy [4, 13, 21]. Given the impact of CMs on cardiac structure and hemodynamics, echocardiography plays a central role in both preoperative evaluation and postoperative follow-up. However, data on echocardiographic characteristics, particularly postoperative changes, remain limited and are primarily derived from small case series [22–25].

Although several studies have explored the prevalence, characteristics, and outcomes of CMs globally, comprehensive data from Iran remain limited, and no studies have been conducted in Isfahan [26–28]. In addition, data on paired preoperative and postoperative echocardiographic parameters are scarce.

This study was therefore designed to evaluate changes in echocardiographic parameters before and after surgical resection of CMs in patients treated in Isfahan over six years, while also characterizing their clinical presentation, histopathological features, tumor location, and anatomical distribution to provide an integrated understanding of their diagnostic and functional implications.

## Methods

### Study design and population

This retrospective cohort study was conducted over 6 years (2018–2023) at a referral cardiac center and a tertiary cardiac surgery center. Given the relative rarity of CMs, a retrospective design was adopted to ensure an adequate sample size could be drawn from available patient records.

The study population comprised all consecutive patients with an CMs confirmed by transthoracic echocardiography (TTE), independently interpreted by two experienced cardiologists.

During the study period, 89 patients with echocardiographically confirmed CMs were identified. Patients were eligible for inclusion if they underwent surgical exploration and excision of the mass. Fourteen patients were excluded due to incomplete hospital records (*n* = 5) or because the lesions were subsequently identified as non-neoplastic entities managed according to alternative clinical pathways rather than surgical mass excision, including infective endocarditis vegetations (*n* = 6) and calcific lesions mimicking tumors (*n* = 3).

**Fig. 1:**
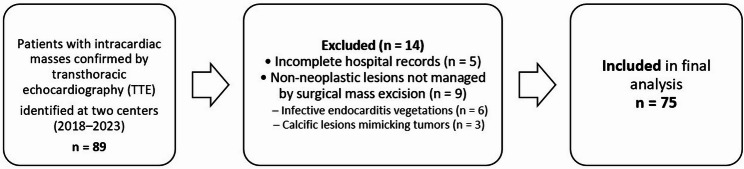
Study flow diagram

The final study cohort, therefore, consisted of 75 patients who underwent complete surgical resection of an CM. Both neoplastic and non-neoplastic lesions (e.g., thrombi) were included if surgically managed as space-occupying CMs. The final diagnosis was established based on histopathological examination of the excised specimens (Fig. 1).

### Study endpoints

The primary endpoint of this study was the evaluation of changes in echocardiographic parameters before and after surgical resection.

Secondary endpoints included clinical presentation, histopathological characteristics of CMs, tumor location, and distribution of tumor types by anatomical location.

### Data collection

The resected specimens were examined histopathologically by a pathologist and classified into four categories: fibrin or blood clots, benign cardiac tumors, malignant cardiac tumors, and metastatic tumors. Tumor classification was performed according to the histopathological criteria outlined in the 2015 World Health Organization classification of cardiac tumors [22].

TTE was performed with the same device by two cardiologists before and after surgical resection. Echocardiographic evaluation included measurement of several parameters, including ejection fraction (EF), mitral regurgitation, mitral stenosis, aortic regurgitation, aortic stenosis, tricuspid regurgitation, pulmonary insufficiency, pericardial effusion, and atrial and ventricular size and function. Preoperative echocardiography was conducted when CMs were suspected, while postoperative echocardiography was performed as part of routine inpatient care prior to hospital discharge.

Clinical data were extracted from hospital records by a cardiac surgery fellow and subsequently verified by a senior cardiac surgeon to ensure data accuracy. The collected data included demographic characteristics (age and sex), patient comorbidities, tumor location, presenting symptoms, and echocardiographic findings before and after surgery.

This study was designed as a retrospective cohort based on medical record data, and no prospective longitudinal follow-up was predefined. At the time of data collection in 2023, all patients were contacted by telephone to assess survival status and tumor recurrence. This information was supplemented by a review of available hospital records when applicable. Outcome status was successfully obtained for all patients (100% ascertainment).

### Ethical considerations

Ethical approval for this study was obtained from the Ethics Committee of Isfahan University of Medical Sciences, under approval number IR.MUI.MED.REC.1402.400. Given the retrospective nature of this study, the need to obtain written informed consent from patients was waived by the Ethics Committee. The study was conducted in accordance with the principles of the Declaration of Helsinki.

### Statistical analysis

This study investigated the prevalence and outcomes of CMs in patients treated at two major cardiac centers in Isfahan over six years. The demographic, clinical, and echocardiographic characteristics of 75 patients were analyzed. Initially, descriptive data are presented as mean ± standard deviation for continuous variables and as frequencies and percentages for categorical variables. Continuous variables were tested for normality using the Kolmogorov–Smirnov test. Normally distributed continuous variables were compared before and after surgery using the paired t-test, while non-normally distributed variables were analyzed using the Wilcoxon signed-rank test. Paired categorical variables were analyzed using McNemar’s test. Statistical analyses were performed using SPSS version 20, and p-values < 0.05 were considered statistically significant.

## Results

### Descriptive findings

#### Baseline characteristics

##### *Patient characteristics*

At the time of study completion in 2023, survival status and tumor recurrence were ascertained for all included patients through direct telephone contact and review of available medical records. No deaths or tumor recurrences were identified among the study population at that time. 

A total of 75 patients with surgically resected CMs were included in the final analysis. The mean age of the cohort was 53.61 ± 17.44 years (range: 2–85 years). The sex distribution was nearly equal, with 38 males (50.7%) and 37 females (49.3%).

Regarding comorbidities, hypertension was present in 9 patients (13.4%), diabetes mellitus in 7 patients (10.4%), and hyperlipidemia in 6 patients (9.0%) (Table 1).

**Table 1:** Demographic factors of patients with cardiac masses

Demographic factors	level	frequency	Percentage (%)
Sex	Male	38	50.7%
	Female	37	49.3%
Comorbidities	Diabetes	7	10.4%
	Hypertension	9	13.4%
	Hyperlipidemia	6	9%

##### *Clinical presentation*

The most common presenting symptom was dyspnea, reported in 33 patients (44%), followed by chest pain (20%) and palpitations (13.3%). Combined symptoms of dyspnea and chest pain were observed in 8% of patients, while syncope occurred in 4%. Rare presentations included dyspnea with palpitations (1.3%) and fever (1.3%). An additional 8% of patients reported other nonspecific symptoms. 

#### Tumor characteristics

##### *Tumor location*

The left atrium was the most common location for CMs, accounting for 48% (*n* = 36) of cases, followed by the right atrium (24%, *n* = 18). Less frequently involved sites included the pericardium (8%, *n* = 6), inferior vena cava (6.7%, *n* = 5), left ventricle (5.3%, *n* = 4), right ventricle (5.3%, *n* = 4), and the aortic valve (2.7%, *n* = 2). 

#### Tumor histopathological characteristics

Histopathological examination revealed that benign tumors represented the majority of CMs (52 patients, 69.3%), whereas malignant tumors were identified in 5 patients (6.7%). Additionally, 18 patients (24%) were diagnosed with fibrin or blood clots.

Among the benign tumors, myxoma was the most frequently observed tumor type, accounting for 39 cases (52% of all CMs). Other benign tumors included cysts (pericardial and hydatid cysts) in 6 cases (8%), leiomyoma in 3 cases (4%), papillary fibroelastoma in 2 cases (2.7%), and hemangioma in 1 case (1.3%).

Five metastatic cardiac tumors were also documented. Among these cases, three were associated with uterine leiomyoma, while one patient had papillary carcinoma originating from the kidney and one patient had lymphoma originating from the adrenal gland (Table 2).

**Table 2:** Histopathological results of cardiac masses

Cardiac mass	Types	*N* = 75 (%)	Total
Fibrin clot	Fibrin clot/Blood clot	18(24%)	18(24%)
Benign cardiac tumors	Myxoma	39(52.0%)	52(69.3%)
	Cysts (pericardial and hydatid cysts)	6(8.0%)	
	Leiomyoma	3(4.0%)	
	Papillary fibroelastoma	2(2.7%)	
	Hemangioma	1(1.3%)	
	Necrotic material with inflammatory infiltration	1(1.3%)	
Malignant cardiac tumors	Spindle cell tumor	1(1.3%)	5(6.7%)
	Papillary carcinoma	1(1.3%)	
	Lymphoma	3(4.0%)	

### Analytical findings

#### Primary endpoint

##### *Echocardiographic parameters before and after surgery*

Paired preoperative and postoperative transthoracic echocardiographic parameters were analyzed to evaluate cardiac functional changes following tumor resection (Table 3). 

Most echocardiographic parameters remained largely unchanged after surgery. However, right ventricular (RV) size demonstrated a statistically significant difference between the preoperative and postoperative assessments (*p* = 0.008). Normal RV size was observed in 98.7% of patients before surgery and 96% after surgery.

Other parameters, including mitral regurgitation, mitral stenosis, aortic regurgitation, aortic stenosis, tricuspid regurgitation, pulmonary insufficiency, and ventricular dimensions, showed minor postoperative variations that did not reach statistical significance (*p* > 0.05).

The mean EF showed a slight decrease after surgery (52.36 ± 7.08 before surgery vs. 51.61 ± 8.77 after surgery), although this difference was not statistically significant (*p* = 0.271).

**Table 3:** Echocardiographic parameters before and after surgery

Parameters	Level	Before	After	*P* value^+^
EF	-	52.36 ± 7.08	51.61 ± 8.77	0.271
MR	No	47(62.7%)	50(66.7%)	0.634
	Mild	21(28%)	20(26.7%)	
	Mild to moderate	5(6.7%)	3(4%)	
	Moderate	2(2.7%)	0	
	Severe	0	2(2.7%)	
MS	No	69(92%)	74(98.7%)	0.073
	Mild	2(2.7%)	0	
	Mild to moderate	1(1.3%)	1(1.3%)	
	Severe	3(4%)	0	
AR	No	65(86.7%)	67(89.3%)	0.456
	Mild	6(8%)	5(6.7%)	
	Mild to moderate	1(1.3%)	0	
	Moderate	2(2.7%)	3(4%)	
	Severe	1(1.3%)	0	
AS	No	74(98.7%)	75(100%)	0.317
	Moderate	1(1.3%)	0	
TR	No	44(58.7%)	46(61.3%)	0.939
	Mild	19(25.3%)	17(22.7%)	
	Mild to moderate	9(12%)	7(9.3%)	
	Moderate	2(2.7%)	5(6.7%)	
	Severe	1(1.3%)	0	
PE	No	73(97.3%)	66(88%)	0.366
	Mild	2(2.7%)	9(12%)	
PI	Normal	56(74.7%)	64(85.3%)	0.999
	Mild	19(25.3%)	11(14.7%)	
LV Size	Normal	75(100%)	74(98.7%)	0.317
	Mild enlargement	0	1(1.3%)	
LV Systolic dysfunction	No	65(86.7%)	61(81.3%)	0.637
	Mild	7(9.3%)	10(13.3%)	
	Mild to moderate	3(4%)	4(5.4%)	
LV diastolic dysfunction	No	64(85.3%)	62(82.7%)	0.257
	Mild	11(14.7%)	8(10.7%)	
	Mild to moderate	0	5(6.6%)	
RV size	Normal	74(98.7%)	72(96%)	**0.008** ^*^^*^
	Mild enlargement	1(1.3%)	2(2.7%)	
	Mild to moderate enlargement	0	1(1.3%)	
RV systolic dysfunction	No	70(93.3%)	59(78.7%)	0.180
	Mild	3(4%)	9(12%)	
	Moderate	2(2.7%)	7(9.3%)	
RV diastolic dysfunction	No	71(94.7%)	74(98.7%)	0.134
	Mild	4(5.3%)	1(1.3%)	

*EF Ejection fraction, *MR* Mitral regurgitation, *MS* Mitral stenosis, *AR* Aortic regurgitation, *AS* Aortic stenosis, *TR* Tricuspid regurgitation, *PE* Pericardial effusion, *PI* Pulmonary insufficiency, *LV* Left ventricle, *RV* Right ventricle

** Statistically significant (p <0.05)

#### Secondary analytical finding

##### *Distribution of tumor types according to location*

The distribution of CM types varied significantly according to tumor location (p < 0.001). Among fibrin clots, the most common site was the right atrium, accounting for 9 cases (50%), followed by the left atrium (22.2%) and left ventricle (16.7%). Smaller proportions were observed in the pericardium (5.6%) and right ventricle (5.6%). 

Benign cardiac tumors were most frequently located in the left atrium, where 32 cases (58.2%) were identified. Other locations included the right atrium (16.4%), pericardium (9.1%), inferior vena cava (7.3%), right ventricle (5.5%), and aortic valve (3.6%).

Malignant cardiac tumors were less common and were identified in the inferior vena cava (50%) and left ventricle (50%) (Table 4).

**Table 4:** Prevalence of sites associated with the type of cardiac mass

	Fibrin clot	Benign cardiac tumors	Malignant cardiac tumors	P value
Aortic valve	0	2(3.6%)	0	< 0.001
Inferior vena cava (IVC)	0	4(7.3%)	1(50%)	
Left atrial	4(22.2%)	32(58.2%)	0	
Left ventricular	3(16.7%)	0	1(50%)	
Pericardial	1(5.6%)	5(9.1%)	0	
Right atrial	9(50%)	9(16.4%)	0	
Right ventricular	1(5.6%)	3(5.5%)	0	

## Discussion

This six-year retrospective study provides insights into the epidemiology and postsurgical echocardiography outcomes of CMs in Isfahan, Iran, and describes paired preoperative and postoperative echocardiographic findings during hospitalization. The mean age of the patients was 53.6 years, with a nearly equal distribution between the sexes. Most patients had benign tumors (69.3%), with myxomas being the most prevalent subtype (52%). The left atrium was identified as the most affected site, accounting for 58.2% of cases.

Postoperative echocardiography was performed as a pre-discharge assessment. Compared with preoperative findings, a statistically significant change in the distribution of RV size categories was observed after surgery (*p* = 0.008). However, the proportion of patients classified as having normal RV size remained high in the postoperative period. Additionally, other parameters showed slight postoperative changes; however, these differences were not statistically significant, possibly because of the small patient sample size.

The results of this study align with those of previous studies on cardiac tumors, particularly regarding tumor prevalence, histopathology, and location. Consistent with global findings, this study confirmed that benign cardiac tumors, especially myxomas (52%), constitute the majority of cases. Global data suggest that benign cardiac tumors represent approximately 70% of all CMs, with myxomas constituting approximately half of cases [29–31]. For example, a meta-analysis reported similar trends, with 68.8% of patients diagnosed with myxomas [29]. Other studies conducted in Western countries reported that 72–73% of cardiac tumors are benign, with half of the cases being myxomas [30, 31]. In Iranian reports, the majority of cases were myxomas, but they reported a higher prevalence: In a five-year single-center study, 84% of PCTswere myxomas; in a 20-year tertiary hospital in the capital, 86% of all tumors were myxomas [26, 28].

Additionally, in another regional study, 73% of cardiac tumors were myxomas [27]. Our findings align more closely with global data and are lower than those of regional studies. This discrepancy may be due to differences in referral patterns or to the broader inclusion criteria used in our study, which led to a larger sample size than in regional studies. Our findings revealed that approximately half of all the masses (48%) and 58.2% of the benign tumors were located in the left atrium, corroborating the international literature. These studies suggest that the left atrium is the most common site for cardiac tumors, particularly PCTs [15, 16, 32–35]. Prior Iranian studies with smaller sample sizes reported that more than 80% of PCTs are located in the left atrium [26–28].

Regarding patients’ symptoms, dyspnea has been identified as the most prevalent clinical manifestation in several studies, a finding consistent with our findings. The reported prevalence of dyspnea varies among populations; non-Iranian studies reported frequencies ranging from 47.1% to 62.5%, whereas Iranian studies documented higher prevalence, ranging from 93% to 100% [25–27, 29, 32, 35–37]. In this study, dyspnea was reported in 44% of patients. The differences between our findings and those of other Iranian studies may be due to narrower inclusion criteria, resulting in smaller sample sizes. Those regional studies focused exclusively on myxoma, nonmyxoma tumors, and PCTsseparately, thus excluding other histopathological types [26–28].

Our postoperative echocardiographic findings showed a slight decrease in the proportion of patients with normal RV size (98.7% vs. 96%), with only 2 patients, one of whom was reclassified as having mild to moderate RV enlargement. Given the small magnitude of this change, its clinical significance is likely limited.

Data describing postoperative RV remodeling after cardiac tumor resection are sparse. Available evidence is largely limited to case reports demonstrating that tumor removal can significantly alter right-sided loading conditions and intracardiac hemodynamics. For example, resection of a RV outflow tract tumor has been associated with marked reductions in right atrial and RV dimensions, the RV Outflow Tract (RVOT)size, and trans-pulmonary gradient, reflecting relief of obstruction [38]. Similarly, surgical removal of a giant RV myxoma resulted in functional recovery consistent with substantial hemodynamic unloading [39].

At a higher level of evidence, a retrospective cohort study of patients undergoing resection of tumor thrombus involving the inferior vena cava also demonstrated an increase in RV size after tumor removal, likely due to abrupt changes in venous return and right-sided loading conditions [40].

In contrast to these conditions involving direct right-sided pathology, the subtle increase in RV size observed in our cohort, predominantly composed of left atrial tumors, may reflect more modest perioperative alterations in RV loading conditions, such as changes in preload, afterload, tricuspid regurgitation severity, or ventricular interdependence. However, no single definitive mechanism can be inferred [41, 42]. These differences highlight that the mechanisms underlying RV changes may vary depending on tumor location and hemodynamic context.

Regarding valvular stenosis, postoperative differences were observed. Specifically, 99% of patients with mitral stenosis and 100% of those with atrial stenosis were classified as having normal findings postoperatively. Nonetheless, these results were not statistically significant, possibly due to the small sample size. Data regarding postoperative echocardiographic changes in patients undergoing cardiac tumor resection remain limited. In patients undergoing tumor thrombus resection involving inferior vena cava, intraoperative echocardiography demonstrated an increase in RV size after tumor removal [40]. Although the magnitude and clinical context differ, our findings are conceptually consistent with this study, suggesting RV size may increase in a subset of patients following tumor removal, particularly in the early perioperative period. These findings underscore the need for further studies in patients undergoing cardiac tumor resection, incorporating both early pre-discharge and long-term echocardiographic follow-up, to better characterize postoperative echocardiographic parameters and their functional implications.

### Strengths and limitations

This study provides novel regional data on CMs from Isfahan, addressing a gap in the literature where data from Iran remain limited [26–28]. A key strength is the inclusion of paired preoperative and postoperative echocardiographic assessments, enabling direct within-patient evaluation of functional changes following surgical resection. In addition, all cases were confirmed by histopathological examination, ensuring diagnostic accuracy. The integration of clinical, echocardiographic, and pathological data provides a comprehensive, clinically relevant evaluation of CMs in real-world practice.

However, several limitations should be considered. First, the retrospective design and single-region setting may limit the generalizability of the findings. Second, given the rarity of cardiac tumors, the relatively small sample size may have reduced the statistical power for certain echocardiographic comparisons. Third, postoperative echocardiographic assessment was limited to the early pre-discharge period, which may not reflect longer-term functional changes and could be influenced by transient postoperative hemodynamic conditions.

Furthermore, some clinical and diagnostic data were not consistently available, including detailed electrocardiographic findings, which were not systematically recorded and therefore could not be reliably analyzed. Although some studies, such as those by Francesco Angeli et al. in a large cohort, have examined electrocardiogram findings [43], similar analysis was not feasible in our study. In addition, advanced imaging modalities such as CMR and computed tomography were not uniformly available, although a 2024 review highlights the important role of multimodality imaging techniques in the diagnosis and management of CMs and outlines future directions for their application [7]. Additionally, the structured diagnostic evaluation model (DEM), proposed to improve the characterization of CMs [12, 44], could not be applied due to incomplete echocardiographic data and its limited integration into routine clinical practice. Finally, outcome assessment was limited to a single time point at data collection, and the absence of systematic longitudinal follow-up precluded robust evaluation of long-term outcomes, including recurrence and survival.

## Conclusion

This study demonstrates that most CMs are benign and that surgical resection is not associated with significant short-term impairment in cardiac function. Postoperative echocardiographic assessment underscores the need for further studies with both early and long-term follow-up to characterize postoperative changes better better. As one of the few studies from Iran incorporating paired pre- and postoperative echocardiographic data with histopathological confirmation, it provides important regional insights. Larger prospective studies are needed to validate these findings and clarify long-term outcomes.

## Data Availability

The datasets generated and/or analysed during the current study are not publicly available due to the inclusion of identifiable and sensitive health information of patients. Public sharing of this data would violate institutional data protection policies and ethical regulations. However, the data are available from the corresponding author upon reasonable request, subject to appropriate safeguards.

## Abbreviations

CMR: Cardiac magnetic resonance
CMs: Cardiac masses
DEM: Structured diagnostic evaluation model
EF: Ejection fraction
PCTs: Primary cardiac tumors
RV: Right ventricle
TTE: Transthoracic echocardiography
